# AN INNOVATIVE EX-VIVO MODEL FOR RAPID CHANGE OF THE PAPILLA FOR TEACHING ADVANCED ENDOSCOPIC RETROGRADE CHOLANGIOPANCREATOGRAPHY PROCEDURES

**DOI:** 10.1590/0102-6720201600040013

**Published:** 2016

**Authors:** Everson L.A. ARTIFON, Thaisa S. NAKADOMARI, Leandro Y. KASHIWAGUI, Emilio A. BELMONTE, Cláudio R. SOLAK, Spencer CHENG, Carlos K. FURUYA, Jose P. OTOCH

**Affiliations:** 1 Postgraduate Program, Departament of Surgery, University of São Paulo, São Paulo, SP, Brazi; 2 GI Endoscopy Fellows - Hospital Sugisawa, Curitiba; Brazi; 3 Animal Lab, IRCAD Latin America, Barretos, São Paulo, SP; Brazi; 4 Departament of Endoscopy, University Hospital of Ponta Grossa, Ponta Grossa, PR, Brazil.

**Keywords:** Endoscopy, Cholangiography, Choledocholithiasis, Biliary protesis

## Abstract

**Background:**

Models for endoscopic retrograde cholangiopancreatography training allow practice with an expert feedback and without risks. A method to rapidly exchange the papilla can be time saving and accelerate the learning curve.

**Aim:**

To demonstrate a newly method of rapid exchange papilla in ex-vivo models to teach retrograde cholangiopancreatography advanced procedures.

**Methods:**

A new model of ex-vivo papilla was developed in order to resemble live conditions of procedures as cannulation, papilotomy or fistula-papilotomy, papiloplasty, biliary dilatation, plastic and metallic stentings.

**Results:**

The ex-vivo model of papilla rapid exchange is feasible and imitates with realism conditions of retrograde cholangiopancreatography procedures.

**Conclusion:**

This model allows an innovative method of advanced endoscopic training.

## RESUMO

**Racional:**

Nas últimas décadas os simuladores de colangiopancreatografia retrógrada tiveram grande evolução. Atualmente dispõem-se de vários métodos para realizar o treinamento minimizando os riscos de complicações e tornando a curva de aprendizagem muito rápida.

**Objetivo:**

Demonstrar uma variação dos modelos ex-vivos desenvolvidos para o treinamento avançado em colangiopancreatografia retrograda.

**Método:**

Foi criado um modelo ex-vivo simulando fatores e condições reais para o treinamento avançado de colangiopancreatografia retrograda como canulação, papilotomia, fistulotomia, papiloplastia e uso de próteses plásticas e metálicas.

**Resultados:**

Esse modelo ex-vivo, com a possibilidade de troca rápida da papila, mostrou-se viável e no treinamento simula condições muito próximas às reais.

**Conclusão:**

Neste modelo há grande inovação para o treinamento de novos endoscopistas em procedimento avançados na colangiopancreatografia retrógrada.

## INTRODUCTION

Several training simulators are currently available for education in diagnostic and interventional endoscopic retrograde cholangiopancreatography (ERCP)^1^ ^,^ ^4^. Models for ERCP training allow practice with expert feedback and without risks.Currently, there are three major categories: virtual reality simulators, live pigs and ex-vivo porcine models^1^. Ex-vivo porcine model offer a low cost training method and yield the possibility of customizing biliary anatomy. A method to rapidly exchange the papilla is essential to save time between the procedures.

The aim of this study was to demonstrate papilla change in ex-vivo models developed for the advanced training in ERCP.

## METHODS

The scenario was allocated in an adequate room structure in order to fit 20 attendees and eight faculties. Were used five ex-vivo scenarios in total. The endoscopic devices were used exclusively to animal lab procedures and according to the sanitary surveillance laws. 

Was used a therapeutic videoduodenoscope and monopolar electrosurgical cautery. Accessories utilized were balloon extraction, basket, biliary dilation balloon, papillotome, needle knife papillotome, 0.035 flexible guidewire, plastic and metal stents.

The ex-vivo model was made from a porcine digestive tract specimen (esophagus, stomach, duodenum, pancreas, liver and gallbladder), which was cleaned and prepared to support the "neo papilla" and "neo choledochus". They were prepared and kept in cold storage 24 h before procedure.

**FIGURE 1:**
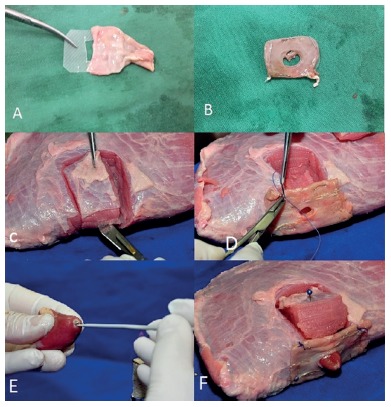
Ex-vivo model structured: A and B) Duodenal window created with a rectangular peace of plastic sheet inserted into small bowel segment; the center hole is settled to pass the chicken heart ("neo papilla"); C) small excavation created into the beef to accommodate the chicken heart; D) duodenal window is fixed in the lateral part of the beef that represents the head of pancreas; E) crochet needle creating the papilla orifice; F) chicken heart inserted in the duodenal window simulating the papilla.

**FIGURE 2:**
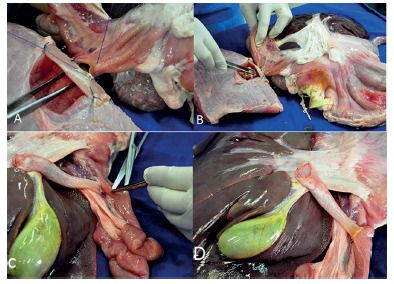
Ex-vivo model structured: A) window with approximately 1 cm in diameter was created in the pig's duodenum and sutured to the duodenal window at the mesenteric border; B) chicken heart inserted in the duodenal window and specimen model completed; C) an esophageal mucosa/submucosa segment mimicking the choledochus in the second portion of duodenum; D) second model (final specimen)

 Firstly, was constructed a duodenal window with a 1x1.5 inch rectangular plastic sheet with a central hole covered by a segment of pig's gut fixed with staples (Figure 1 A and B). A piece of beef with an excavation in the lateral side was made (Figure 1C) and sutured to the duodenal window (Figure 1D). This window was then sutured to an opening in the duodenal wall to accommodate the "neo papilla" and allow its rapid exchange (Figure 2A). The piece of beef simulates the head of the pancreas and grants stability during the procedures. To resemble the papilla we used a chicken heart. In the apex of the chicken's heart a tunnel was created to mimetize the ampulla (Figure 1E). The chicken heart was accommodated in excavation and inserted into duodenal window (Figure 1F and 2B). The second model demonstrate stone extraction, metal and plastic stenting. In this model was constructed an artificial choledochus by using a segment of the esophageal mucosa/submucosa, connecting the liver to the duodenum (Figure 2 - C and D). An artificial stenosis was created with piece of elastic. This elastic is tied externally.

Both models are manufactured once and after each cannulation, papillotomy, fistulopapillotomy or papilloplasty the chicken´s heart is exchanged, allowing a rapid reset of local anatomy for a new procedure.

The ex-vivo model was fitted into a plastic mannequin and the esophagus attached to a plastic tube that was placed at the mannequin mouth. The duodenoscope was inserted in the second portion of the duodenum and left in place for the procedure.

The papilla and the choledochus model were structured and prepared to performing the following advanced ERCP procedures: Model 1 for cannulation and papillotomy; Model 2 for fistulotomy; Model 3 for papilloplasty; Model 4 for biliary stone extraction; and Model 5 for plastic and metal stenting.

## RESULTS

### Model 1: Cannulation and papillotomy

**FIGURE 3:**
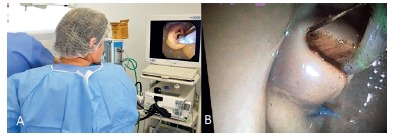
A) Image showing trainee performing a papillotomy; B) endoscopic view of the procedure

Trainees were able to use flexible guidewire to cannulate "the neo papilla" and perform papillotomies. After the completion of procedures, the chicken heart was changed to the next student (Figure 3 - A and B). 

### Model 2: Fistulotomy

**FIGURE 4:**
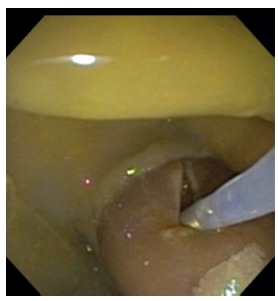
Image demonstrating needle knife fistulotomy

Students performed a papillary fistulotomy by using a needle knife catheter, a guide wire and a cut thermic current. The procedure was made under supervising of an experienced faculty (Figure 4).

### Model 3: Papilloplasty

**FIGURE 5:**
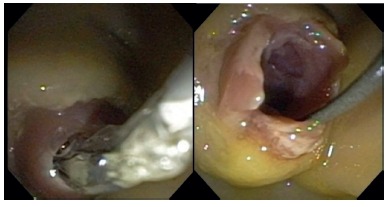
Images demonstrating the balloon in position and papilloplasty after dilation

Trainees were able to use a dilation balloon to perform papilloplasty and enlarge the diameter of the papilla (Figure 5).

### Model 4: Biliary stone extraction

**FIGURE 6:**
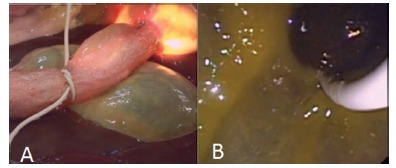
A) Image showing a biliary stricture; B) image demonstrating a "stone" been removed

For this procedure it uses the proposed model (artificial bile duct). In the middle third of this "common bile duct" it is created an artificial stenosis with an elastic. In this model the student can perform the biliary balloon dilatation, and then perform the "stone" (coffee beans) extraction with extractor ballon (Figure 6 - A and B).

### Model 5: Plastic and metal stenting

**FIGURE 7:**
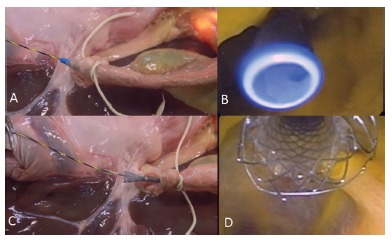
A and B) Images demonstrating plastic stenting; C and D) images showing metal stenting placement

In this station, the students were capable of using a flexible guidewire, plastic and metal stent to drain the biliary duct. To this procedure were performed in both models, with chicken's heart or neo choledochus. If the monitor chose the chicken's heart model, the student needed to perform the catheterization, papillotomy, fistulotomy or papilloplasty and after then plastic or metallic stenting (Figure 7 - A, B, C and D).

## DISCUSSION

Education in ERCP involves the development of clinical making, imaging interpretation, and technical skills. Access to simulator training is attractive by virtue of opportunity for risk-free practice with expert feedback^1^ ^,^ ^4^. There are three main training models: virtual reality simulators, live pig and ex-vivo porcine models. 

The first one is very expensive and has other disadvantage: the lack realism. The method is less productive after 20 to 50 procedures, so that is more useful as training in basic navigation. 

The animal models provide better realistic experience, but there are some disadvantages: is expensive, needs a specific infrastructure, and has ethical concerns with its use. Also has two others limitations: the location of native papilla and the natural orifice of the pancreatic duct is located more distally in the duodenum^1^ ^,^ ^4^ ^,^ ^5^. 

Ex-vivo porcine models offer the advantages of realism, are easier to use, are less costly, and eliminate ethical concerns. Artifon et al^1^ ^,^ ^2^ demonstrated the feasibility of ex-vivo models. Some ex-vivo porcine model uses the native papilla, but, as said, it has some limitations: the small size of the papilla, its location, and the impossibility of making several sphincterotomies in the same model.^1^

Due to these difficulties, some authors have developed models using chicken´s heart to simulate the duodenal papilla (neo papilla)^5^ ^,^ ^6^ ^,^ ^7^. But this model takes a long time to prepare, because the papilla (chicken hearts) is sutured into the porcine stomach^6^. While models, like Matthes^4^, take approximately 75 min to be produced our model has some advantages. First, it can be reproduced with easy way, and the papilla can be placed anatomically and can be quickly exchanged.

## CONCLUSION

In conclusion, this new model is cheap, reproducible, realistic and adds agility during the teaching process allowing the rotation of a lot of attendees.
